# Bax/Mcl-1 balance affects neutrophil survival in intermittent hypoxia and obstructive sleep apnea: effects of p38MAPK and ERK1/2 signaling

**DOI:** 10.1186/1479-5876-10-211

**Published:** 2012-10-22

**Authors:** Larissa Dyugovskaya, Andrey Polyakov, Victoria Cohen-Kaplan, Peretz Lavie, Lena Lavie

**Affiliations:** 1 The Lloyd Rigler Sleep Apnea Research Laboratory, The Ruth and Bruce Rappaport Faculty of Medicine, Technion-Israel Institute of Technology, Haifa, Israel; 2 Cancer and Vascular Biology Research Center, The Ruth and Bruce Rappaport Faculty of Medicine, Technion-Israel Institute of Technology, Haifa, Israel; 3 Unit of Anatomy and Cell Biology, The Ruth and Bruce Rappaport Faculty of Medicine, Technion, POB 9649, Haifa, 31096, Israel

**Keywords:** Intermittent hypoxia, Obstructive Sleep Apnea, Neutrophil mitochondrial apoptotic pathway, Bax/Mcl-1 balance, ERK 1/2 signaling, p38MAPK signaling

## Abstract

**Background:**

Prolonged neutrophil survival is evident in various cardiovascular and respiratory morbidities, in hypoxic conditions *in-vitro* and in patients with obstructive sleep apnea (OSA) characterized by nightly intermittent hypoxia (IH). This may lead to persistent inflammation, tissue injury and dysfunction. We therefore investigated by a translational approach the potential contribution of the intrinsic stress-induced mitochondrial pathway in extending neutrophil survival under IH conditions. Thus, neutrophils of healthy individuals treated with IH *in-vitro* and neutrophils of OSA patients undergoing nightly IH episodes *in-vivo* were investigated. Specifically, the balance between pro-apoptotic Bax and anti-apoptotic Mcl-1 protein expression, and the potential involvement of p38MAPK and ERK1/2 signaling pathways in the control of Mcl-1 expression were investigated.

**Methods:**

Purified neutrophils were exposed to IH and compared to normoxia and to sustained hypoxia (SH) using a BioSpherix-OxyCycler C42 system. Bax and Mcl-1 levels, and p38MAPK and ERK1/2 phosphorylation were determined by western blotting. Also, Bax/Mcl-1 expression and Bax translocation to the mitochondria were assessed by confocal microscopy in pre-apoptotic neutrophils, before the appearance of apoptotic morphology. Co-localization of Bax and mitochondria was quantified by LSM 510 CarlZeiss MicroImaging using Manders Overlap Coefficient. A paired two-tailed *t test,* with Bonferroni correction for multiple comparisons, was used for statistical analysis.

**Results:**

Compared to normoxia, IH and SH up-regulated the anti-apoptotic Mcl-1 by about 2-fold, down-regulated the pro-apoptotic Bax by 41% and 27%, respectively, and inhibited Bax co-localization with mitochondria before visible morphological signs of apoptosis were noted. IH induced ERK1/2 and p38MAPKs phosphorylation, whereas SH induced only p38MAPK phosphorylation. Accordingly, both ERK and p38MAPK inhibitors attenuated the IH-induced Mcl-1 increase. In SH, only p38MAPK inhibition decreased Mcl-1 expression. Similar to neutrophils of healthy subjects exposed to IH (0.97± 0.2), in OSA neutrophils, Bax/Mcl-1 ratio was significantly lower compared to normoxic controls (1.0±0.5 vs.1.99±0.3, p=0.015), and Bax did not co-localize with mitochondria.

**Conclusions:**

These findings suggest that decreased Bax/Mcl-1 balance promotes neutrophil survival in IH *in-vitro* as well as in OSA patients. Moreover, Bax/Mcl-1 protein function in IH and SH might be regulated by different signal transduction pathways, highlighting a novel regulatory function through ERK1/2 signaling in IH.

## Background

Neutrophils are bone marrow derived short-lived cells which provide a unique model to study survival signaling. Once released into the circulation, neutrophils undergo constitutive apoptosis. However, their lifespan is prolonged in coronary syndromes such as unstable angina and acute myocardial infarction and in respiratory diseases such as chronic obstructive pulmonary disease (COPD) and neonatal and adult respiratory distress syndrome (RDS) [1-4]. Prolonged neutrophil survival is also evident in patients with obstructive sleep apnea (OSA), characterized by repeated nightly episodes of intermittent hypoxia (IH) [5]. Of note, increased neutrophil survival within tissues or in the circulation can promote persistent inflammation resulting in tissue injury and dysfunction.

Unlike in other cells, sustained hypoxia (SH) as well as IH were shown to profoundly inhibit neutrophil apoptosis *in-vitro*[5-9] and *in-vivo*[5,10]. Specifically in SH several signaling pathways and a number of family molecules that regulate apoptosis are activated. B-cell lymphocytic-leukaemia proto-oncogene (Bcl)-2 family members are one such family, which can be either pro-apoptotic (Bax and Bak, as well as BH3-only proteins, Bid, Bim, Bad, Bik, Noxa, PUMA) or anti-apoptotic (Mcl-1, Bcl-2, Bcl-X and A1) [11-14]. The Bcl-2 family members are integrated in cell functions at the level of the mitochondria and participate in the regulation of stress-induced apoptosis [15-17].

Bcl-2 associated X protein (Bax) is necessary for inducing apoptosis [18] and its translocation and redistribution to the mitochondria is essential for implementing the apoptotic program [15,18,19]. Therefore, Bax is considered a quantitative marker of early apoptotic events [13,16,20-22]. Anti-apoptotic stimuli inhibit Bax insertion into the mitochondrial membrane, thereby inhibiting its pro-apoptotic activity [13]. On the other hand, myeloid cell leukemia 1 (Mcl-1) promotes neutrophil survival by binding and sequestering Bak and Bax, which are capable of forming pores in the mitochondrial membrane [23]. Mcl-1 is up-regulated in response to various survival stimuli [23-29] and is required for neutrophil viability under SH [30,31]. Importantly, decrease in Mcl-1 levels precedes the appearance of the apoptotic morphology [32].

MAPKs, in particular p38 [33-35] and ERK [36,37] regulate the apoptotic program in neutrophils. Specifically, Mcl-1 expression might be regulated by signal transduction through ERK [28]. ERK is also responsible for increasing Mcl-1 through protein stabilization by granulocyte-macrophage colony-stimulating factor (GM-CSF) [25]. Sustained hypoxia can increase neutrophil survival by activating p38MAPK signaling, thereby inducing Mcl-1 proteins [30].

Previously we have shown that NF-κB, its downstream gene IL-8, CXCR2 receptor expression, and p38MAPK signaling pathways are essential for controlling neutrophil survival in healthy individuals treated with IH *in-vitro* via the extrinsic pathway which is Fas receptors and TNF-α dependent [9]. To further elucidate the mechanisms involved in prolonging neurtophil survival under IH *in-vitro* as well as in patients with OSA, herein we investigated the intrinsic stress-induced mitochondrial pathway. These effects of IH were investigated during the early pro-apoptotic events, which occurred in neutrophils before the appearance of morphological changes and caspase’s cascade activation. Thus, we show that Bax expression was decreased and its translocation to the mitochondria was inhibited under IH *in-vitro*. Concurrently, Mcl-1 expression was up-regulated via activation of ERK1/2 and p38MAPK dependent signaling pathways. Finally, we ascertained the involvement of the mitochondrial network in prolonging the survival of neutrophils in patients with OSA. Similar to the IH *in-vitro* model*,* in OSA patients which represent an IH *invivo* model, Bax did not co-localize with the mitochondria and Bax/Mcl-1 ratio was significantly lower than in healthy controls.

## Methods

### Neutrophil isolation and treatment

Blood samples were obtained from 10 healthy volunteers (age=35.8±11.9 yr, BMI=25.3±2.6 Kg/m^2^) and from 7 OSA patients (age=51.4±15.4 yr, BMI=30.2±5.5 Kg/m^2^, apnea-hypopnea index (AHI)=35.7Â±20 events/hrs). All control subjects and OSA patients were free from cardiovascular disease or diabetes and had normal blood pressure values (not higher than 140/90 mm Hg). All controls and most OSA patients did not take medications for at least 2 weeks before the study was conducted. Two OSA patients used irregularly low-dose acetyl salicylic acid (micropirin-75). In 7/10 healthy-controls, AHI (2.1±1.8 events/hrs) was determined by a validated home monitored device (watchPAT-100 Itamar Medical, Caesarea, Israel) [38] and 3/10 controls underwent full-night polysomnography (AHI 8.0±1.7 events/hrs) as all OSA patients (Technion Sleep Medicine Center, Haifa). OSA diagnosis was based on the recommendations of the American Academy of Sleep Medicine Task Force with a cutoff point of AHI≥10 [39]. Lipid profile and high sensitivity C-reactive protein (CRP) were determined in patients and controls as previously described [5]. The protocol was approved by the local Human Rights Committee, and all participants signed an informed consent form.

Blood samples were withdrawn under fasting conditions and polymononuclear cells (PMNs) were isolated using a two layer Ficoll-Histopaque density gradient centrifugation (Histopaque 1.077 and 1.119, Sigma-Aldrich, Inc., St. Louis, MO, USA). PMN purity was greater than 96%, and viability was greater than 99%, as determined by trypan blue exclusion. Purified PMNs were resuspended in RPMI-1640 medium, supplemented with 10% FCS and 1 mM L-glutamine, plated without/with inhibitors and exposed to normoxia, SH or IH using the BioSpherix-OxyCycler C42 system as we described previously [5,9].

### Light-microscopy assessment of neutrophil apoptosis

Purified neutrophils’ cytospin preparations were fixed, and stained with May Grunwald-Giemsa. Slides were read blindly by Axiovert 25 (Zeizz) light microscope. At least 300 cells/slide were analyzed. Cells showing apoptotic morphology were identified according to the following criteria: nuclear condensation in the form of a single nucleus or nuclear fragments not connected by strands [40,41].

### *In*-*vitro* IH and SH protocol

Purified PMNs (0.6 ml per well; 3–4 × 10^6^ cells/ml) were plated into 24 well plates and then were exposed to normoxia, SH or IH in custom-designed incubation chambers which are attached to an external O_2_-CO_2_-N_2_ computer-driven controller using BioSpherix-OxyCycler-C42 system (Redfield, NY, USA). This system which enables to create periodic changes in external O_2_ concentrations that control air gas levels in each chamber individually was described in detail previously [5,9]. Briefly, for IH, the O_2_ saturation in the medium was kept at 2% for 6.6 ± 3.6 min durations, out of each 1 hr cycle. In each experiment 6 IH cycles were run. SH was employed for a comparable time at 2% actual oxygen in the medium for the entire period. Control purified PMNs were maintained in normoxic conditions for the same durations. Oxygen levels in the medium were determined by a fiber-optic dissolved oxygen electrode (BioSpherix, Redfield, NY, USA).

### Western blot analysis

PMNs cultured in normoxia, IH or SH, were lysed in Tris buffered Saline Triton -X (TBST) at pH 7.4 (50 mM Tris–HCl, 150 mM NaCl, 0.5% Triton X-100, 0.2 mM sodium vanadate), supplemented with a mixture of protease inhibitors (Roche Diagnostics GmbH, Roche Applied Science, Mannheim, Germany), and stored at −80°C until use. Cell lysates were centrifuged at 16,000 × *g* for 15 min and protein concentration was determined by Bradford reagent (Bio-Rad Laboratories GmbH, Munich, Germany). Cell lysates (50 μg) were run on 12% SDS-PAGE and transferred onto Hybond nitrocellulose membranes (Amersham Biosciences Europe GmbH, Freiburg, Germany). Membranes were blocked and incubated with primary rabbit polyclonal antibodies against Thr180/Tyr182-phosphorylated p38MAPK (Cell Signaling Technology, Inc., Beverly, MA, USA), Thr202/Tyr204-phosphorylated ERK1/2 (Cell Signaling Technology, Inc., Beverly, MA, USA), Bax (N20, sc493) and Mcl-1 (S-19, sc819, Santa Cruz Biotechnology Inc., CA, USA), followed by goat anti-rabbit IgG incubation (Amersham Biosciences Europe GmbH, Freiburg, Germany). Then membranes were washed six times with TBST buffer and incubated with horseradish peroxidase-conjugated secondary antibody (goat anti-rabbit IgG; Amersham Biosciences Europe GmbH, Freiburg, Germany) for 1 hr at room temperature. Densitometric analysis was performed using TotalLab TL100 v.2006c software (Nonlinear Dynamics Ltd., Newcastle Upon Tyne, UK), and is expressed in arbitrary units.

### Confocal laser scanning microscopy

Viable neutrophils were stained with 100 nM MitoTracker Orange CMTMRos (Invitrogen, Molecular Probes, Eugene, Oregon, USA) for mitochondria [42]. Then fixed, Triton-permeabilized and labeled with anti-Bax (N20, sc493) or anti-Mcl-1(S-19, sc819) polyclonal antibodies (Santa Cruz Biotechnology Inc., CA, USA) followed by Cy^TM^2-conjugated Goat anti-Rabbit IgG incubation (Jackson ImmunoResearch Laboratories, Inc., Baltimore Pike, PA, USA). Nuclei were stained with TO-PRO-3 (Invitrogen Inc., Carlsbad, CA, USA). Slides were mounted with fluorescence mounting medium (Vectashield H-1000, Vector lab Inc., Burlingame, CA, USA) and were analyzed by confocal laser scanning fluorescence system (Radiance 2000) with Nikon E600 (Japan) camera. Controls for staining included a primary nonspecific rabbit IgG, secondary antibodies and five-fold excess Mcl-1 blocking peptide (sc819 P, Santa Cruz Biotechnology Inc., CA, USA).

### Quantitative fluorescence intensity and co-localization analysis

Relative quantitation of green (Bax) and red (Mitochondrial staining) fluorescence of each cell was accomplished by acquiring grayscale images and fluorescence intensities were integrated using ImageJ 1.42q (Wayne Rasband National Institute of Health, USA). Co-localization of Bax and mitochondria was quantified by LSM 510 CarlZeiss MicroImaging GmbH v.4.2 R&D in collaboration with EmBl, Heidelberg, Germany using Manders Overlap Coefficient (MOC) [43]. Only neutrophils with MOC>0.6 were considered as cells with significant co-localization. At least 50 pre-apoptotic neutrophils from different fields were counted in each sample.

### Inhibitor experiments

MAPK inhibitors included: U0126 (10μM) for MEK1/2 blocking (Signal Transduction, Beverly, MA, USA) and SB202190 (30μM) for p38MAPK blocking (Calbiocem, EMD Chemicals, Inc., NJ, USA) [25,44].

### Statistical analysis

Data are expressed as mean ± SD. A paired two-tailed *t test* was used for single comparison of parametric data. Values of p<0.05 were considered significant. A paired two-tailed *t test* with Bonferroni correction was used to compare the effects of IH and SH vs. normoxia. Therefore, for multiple comparisons only values of p<0.017 were considered significant. The NCSS 2004 statistical package, Kaysville, Utah, USA was used.

## Results

### IH attenuates Bax translocation to the mitochondria and its levels

To determine the effects of IH on neutrophil survival, apoptosis was quantified morphologically by light microscopy. The percentage of apoptotic neutrophils, as determined by a single nucleus with dense chromatin condensation, or nuclear fragments not connected by strands, was 25.0±6.3% in normoxia. Exposing neutrophils to 6 IH cycles or to 6 hrs of SH significantly decreased the percentage of apoptotic neutrophils (14.5±6.5%, p=0.0002 IH vs. normoxia; 19.5±5.6%, p=0.016 SH vs. normoxia). These baseline values confirmed our earlier findings that IH *in-vitro* increased neutrophil survival [5,9].

**Figure 1:**
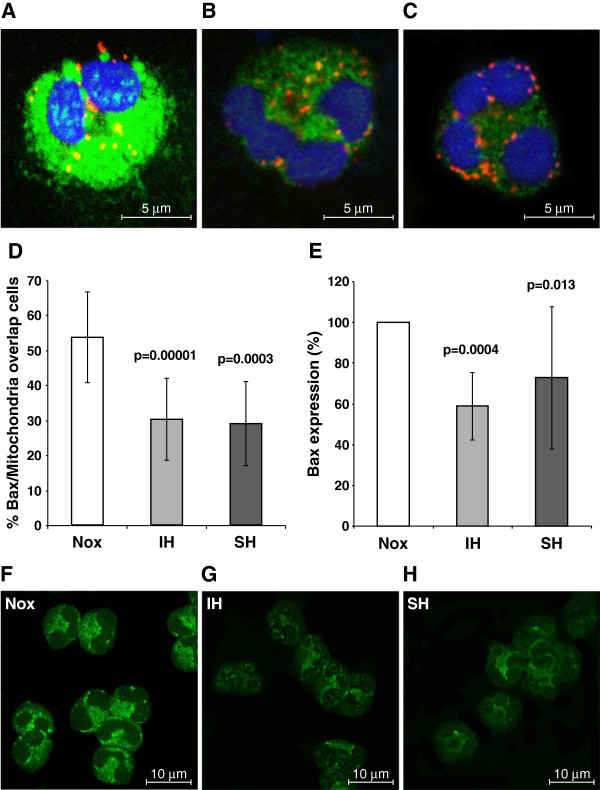
**Bax expression and co-localization with mitochondria in neutrophils exposed to various oxygen conditions.** Bax expression and co-localization with mitochondria were analyzed by confocal laser scanning microscopy as described in methods. The cytoplasmic distribution of Bax (green fluorescence) and mitochondria (red fluorescence) was studied by double immunofluorescence labeling. (**A**) A representative photomicrograph of an apoptotic neutrophil with a very high Bax expression (140,000 arbitrary units) and intensive fusion of Bax with mitochondria (yellow), Manders Overlap Coefficient (MOC)>0.6. (**B**) A pre-apoptotic neutrophil with intensive Bax expression (17,000 arbitrary units). Bax is translocated and co-localized with mitochondria (yellow and orange), MOC>0.6 representing neutrophils predominantly seen at normoxia (Nox). (**C**) A pre-apoptotic neutrophil with low Bax expression (9,500 arbitrary units). Bax (green) and mitochondria (red) are located separately (MOC<0.6), representing neutrophils predominantly seen in intermittent hypoxia (IH) and sustained hypoxia (SH). (**D**). The percentage of pre-apoptotic neutrophils with fusion of Bax and mitochondria (MOC>0.6) in Nox, IH and SH. Values represent the means ± standard deviations (n=10). P values represent significance of IH or SH vs. Nox. (**E**) Relative percentage of Bax expression, assessed by confocal laser scanning microscopy (n=10). The average fluorescence intensity unit per cell, detected by immunofluorescent quantitation at normoxia was considered as baseline (100%), and the effects of hypoxia were calculated as relative decrease of Bax expression. P values represent significance of IH or SH vs. Nox. (**F**-**H**) Representative images of Bax expression (green) out of ten experiments in Nox (**F**), IH (**G**), and SH (**H**).

Under confocal microscopy apoptotic neutrophils were identified by the typical morphology of dense nuclei. The apoptotic neutrophils were also characterized by a very high Bax expression (green fluorescence, from 120,000 to 140,000 units), and its fusion with mitochondria (MOC>0.6; yellow-orange dots), as depicted in Figure 1A. Such apoptotic neutrophils, which are more prevalent in normoxia, were not investigated in further experiments, since we focused on earlier mechanisms that trigger the apoptotic program before visible signs of apoptosis can be detected. Therefore Bax expression and its translocation to the mitochondria under the three oxygen conditions were examined only in pre-apoptotic neutrophils, characterized by normal nuclear morphology (Figure 1B,C).

Neutrophils of healthy subjects were exposed to IH and compared by quantitative immunofluorescence to those exposed to SH and normoxia. In normoxia, pre-apoptotic neutrophils demonstrated intensive fusion of Bax with mitochondria, with a shift in fluorescence to yellow-orange (MOC>0.6), as depicted in Figure 1B. In contrast, in IH and SH treated neutrophils Bax and mitochondria were located separately with diffuse Bax distribution (green fluorescence) and the mitochondria remained dotted (red fluorescence) in the cytoplasm (Figure 1C). MOC in IH and SH treated neutrophils was lower than <0.4, indicating that less than 40% of both components overlapped. Figure 1D summarizes the translocation of Bax and its co-localizion with mitochondria for 10 separate experiments. In normoxia, Bax translocation/co-localization (MOC>0.6) was noted in 53.7±12.9% of the neutrophils. In contrast, after treatment with IH or SH, the percentage of neutrophils with Bax translocating to the mitochondria was significantly decreased as compared to normoxia. However, it did not differ significantly between IH and SH treatments. Bax expression under the three oxygen conditions is summarized in Figure 1E. Since the average fluorescence intensity of Bax expression per cell varied depending on the blood donor investigated, Bax expression in normoxia in each subject was considered as 100%, and the changes induced by IH or SH were plotted as a relative percentage of this value. The cumulative data (Figure 1E) show that Bax expression was significantly down-regulated in neutrophils treated by either IH (by 41%) or SH (by 27%), compared to normoxia. Figure 1F-H depicts representative confocal microscope photomicrographs of Bax expression in normoxia, IH, and SH. After 6 hrs of normoxia the intensity of Bax expression in pre-apoptotic neutrophils was slightly and non-significantly increased by 12% as compared to Time 0.

**Figure 2:**
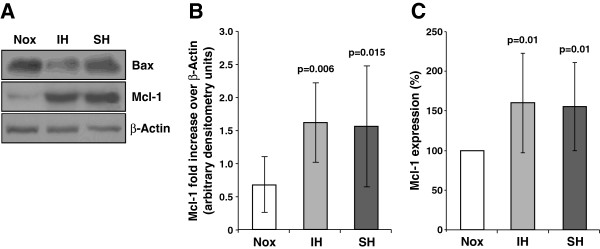
**Mcl-1 expression in neutrophils exposed to various oxygen conditions.** (**A**) A representative western blot depicting Bax and Mcl-1 expression in normoxia (Nox), intermittent hypoxia (IH) and sustained hypoxia (SH) in one out of six experiments performed. (**B**) The normalized values of Mcl-1, obtained by densitometric analysis of western blots are presented as arbitrary units, and depicted as fold induction over β-actin in Nox, IH and SH. (**C**) Mcl-1 expression was assessed by confocal laser scanning microscopy (n=8). The average fluorescence intensity unit per cell, detected by immunofluorescence quantitation at normoxia was considered as baseline (100%), and the effects of IH and SH were calculated as relative Mcl-1increase. P values represent significance of IH or SH vs. Nox.

The decrease in Bax expression in the hypoxic conditions was also confirmed by protein levels as determined by western blot analysis. The following relative values for Bax expression over β-actin were obtained: normoxia, 1.82±0.7 units; IH, 0.96±0.2 units (p=0.03 vs. normoxia) and SH, 0.97±0.5 units (p=0.04 vs. normoxia). A representative immunoblot of Bax protein levels over β-actin from 6 independent experiments is depicted in Figure 2A.

### IH up-regulates the levels of Mcl-1 protein

**Figure 3:**
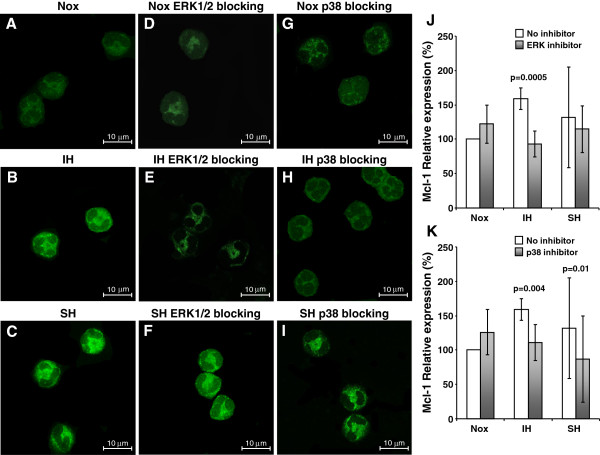
**Effects of ERK and p38MAPK inhibition on Mcl-1 expression in various oxygen conditions.** Neutrophils were incubated for 6 hrs at normoxia (Nox), sustained hypoxia (SH), or 6 cycles of intermittent hypoxia (IH) without or with 10 μM U0126 (a specific inhibitor of MEK1/2, which blocks ERK1/2 activation), and 30 μM SB202190 (a competitive inhibitor of the p38 kinase). Mcl-1 expression was assessed by confocal laser scanning microscopy. (**A**-**I**) Representative photomicrographs of Mcl-1 expression (green) from 1 out of 4 experiments performed. (**A**) Neutrophils incubated in Nox, (**B**) IH and (**C**) SH. (**D**) Neutrophils incubated in Nox with MEK1/2 inhibitor, (**E**) in IH with MEK1/2 inhibitor, and (**F**) in SH with MEK1/2 inhibitor. (**G**) Neutrophils incubated in Nox with p38MAPK inhibitor (**H**) in IH with p38MAPK inhibitor, and (**I**) in SH with p38MAPK inhibitor. (**J**) The average values of Mcl-1 expression for ERK1/2 inhibitor and (**K**) for p38MAPK inhibitor by relative percent. The intensity of Mcl-1 expression at normoxia without inhibitors was considered as 100%, and the changes induced by the IH and SH with or without the inhibitors were plotted as a relative percentage of this value. P values represent significance of untreated vs. inhibitor treated neutrophils in each oxygen condition.

Similar to Bax, total Mcl-1 expression was also assessed at the protein level by western blotting as illustrated in Figure 2A. The average densitometric analysis from 6 independent experiments is presented in Figure 2B. In neutrophils exposed to IH or to SH, Mcl-1 protein fold-increase over β-actin was significantly higher by about 2-fold compared to normoxia. Also Mcl-1 up-regulation was observed by confocal microscopy, as illustrated in Figure 2C. Similar to Bax analysis, the intensity of Mcl-1 expression in normoxia for each subject was considered as 100% and the changes induced by IH or SH were plotted as a relative percentage of this value. The specificity of Mcl-1 was confirmed using five-fold excess of the Mcl-1 blocking peptide, which abolished Mcl-1 fluorescent staining. Representative confocal microscope photomicrographs of Mcl-1 expression in normoxia, IH, and SH are presented in Figure 3A-C. After 6 hrs of normoxia the intensity of Mcl-1 expression in pre-apoptotic neutrophils was decreased by about 20% compared to Time 0.

### Effects of ERK and p38MAPK inhibition on Mcl-1 expression

MAPKs, including ERK1/2 and p38MAPK control neutrophil survival under certain conditions [27,28,36,44,45]. Specific p38MAPK and ERK1/2 inhibitors were used to determine whether Mcl-1 expression was dependent on the activation of MAPK signaling pathways under IH. Neutrophils were incubated in normoxia, SH or IH (as described in methods) with or without 10 μM U0126 (a specific inhibitor of MEK1/2, blocks ERK1/2 activation) or 30 μM SB202190 (a competitive p38MAPK inhibitor). Mcl-1 distribution was determined in pre-apoptotic neutrophils by confocal laser scanning microscopy. Representative images (from 1 out of 4 experiments performed) of Mcl-1 expression in the three oxygen conditions without or with the inhibitors are presented in Figure 3A-I. Figures 3-K depict the average values of Mcl-1 expression for ERK1/2 inhibitor and p38MAPK inhibitor by relative percent when normoxia without the inhibitor was considered as 100%. Blocking ERK/MEK activity slightly increased (p=0.16) Mcl-1 expression in normoxia but significantly decreased the IH-mediated Mcl-1 up-regulation by 40%. In contrast, in SH, Mcl-1 expression was not affected by the ERK/MEK inhibitor (Figure 3D-F, J). Inhibiting p38MAPK also slightly increased (p=0.1) Mcl-1 expression in normoxia, but the hypoxia-induced enhanced Mcl-1 expression, was significantly attenuated in both IH (by 30%) and SH (34%) conditions (Figure 3G-I K ,).

### ERK and p38MAPK activation in response to hypoxia

**Figure 4:**
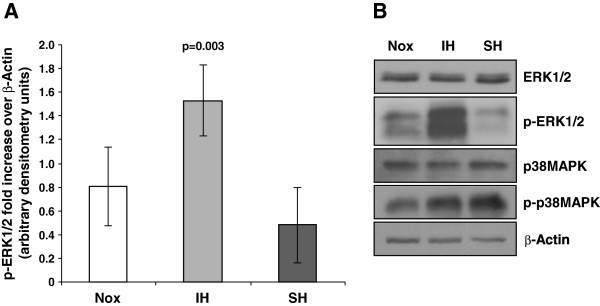
**ERK1/2 and p38MAPK phosphorylation in neutrophils exposed to various oxygen conditions.** (**A**) The average (± SD) normalized values of p-ERK1/2 (n=6), obtained by densitometric analysis of western blots, are presented as fold increase over β-actin in arbitrary units in normoxia (Nox), intermittent hypoxia (IH) and sustained hypoxia (SH). (**B**) A representative western blot depicts the dual phosphorylated (Thr202/Tyr204) form of ERK1/2 (p-ERK1/2) and dual phosphorylated (Thr180/Tyr182) form of p38MAPK (p-p38) compared to non-phosphorylated ERK1/2 and p38MAPK in Nox, IH, and SH.

To directly asses ERK1/2 and p38MAPK activation by IH, their phosphorylation was determined by western blotting. As depicted in Figure 4A, only IH but not SH significantly triggered the phosphorylation of ERK1/2. This pattern of ERK1/2 activation was consistently seen in each separate experiment performed with neutrophils isolated from 6 different donors. For comparison with ERK1/2, we also confirmed our earlier findings showing that p38MAPK phosphorylation was induced in response to both IH and SH [9]. Figure 4B is a representative immunoblot depicting ERK1/2 and p38MAPK phosphorylation. Non-phosphorylated controls of ERK1/2 and p38MAPK did not differ between the treatments.

### Bax expression and co-localization in neutrophils of OSA patients

Bax expression and translocation to the mitochondria was also assessed in neutrophils of OSA patients. Neutrophils cultured for 6 hrs in normoxia or 6 cycles of IH were compared to controls. Three out of seven studied patients were obese having a BMI>30. Three out of ten healthy controls were investigated concurrently with the OSA patients. All underwent full-night polysomnography after which blood samples were taken. Individual demographic, blood chemistry and sleep data for OSA patients and the controls are presented in Table 1.

**Table 1:** Demographic, blood chemistry and sleep apnea measures for patients and controls participating in the experiments

**No**	**Age**	**BMI**	**AHI**	**% TO Sat <90%**	**TG (mg/dL)**	**Cholest. (mg/dL)**	**HDL (mg/dL)**	**LDL (mg/dL)**	**Glucose (mg/dL)**
1 OSA	62	25.2	22	17.3	203.9	183	26.8	115.6	87.2
2 OSA	64	27.4	19	2.2	50.5	215	81	124.3	88.3
3 OSA	48	34.2	33	6.8	79.9	154	60.4	77.5	95.9
4 OSA#	74	35.9	61	50.2	116.7	118	46.3	48.4	71.3
5 OSA*#	42	27.1	15	21.5	80.6	188	35.7	136.2	102.3
6 OSA	33	37.6	65	45.1	217.2	145	28.8	72.8	105.9
7 OSA	37	24.7	35	1.9	82.7	161	48.3	96	91.8
**Mean**	**51.4**	**30.2**	**35.7**	**20.7**	**118.8**	**166**	**46.8**	**95.8**	**91.8**
**SD**	**15.4**	**5.5**	**20.0**	**19.9**	**65.7**	**32.0**	**19.2**	**31.5**	**11.4**
1 Cont.	57	23.4	9	0	123.7	193	51.5	116.6	87.1
2 Cont.	38	25.3	9	0	94.2	185	59	107.2	90.7
3 Cont.	57	25.9	6	0	295.0	227	28.3	140.1	89.2
**Mean**	**50.7**	**24.9**	**8**	**0**	**171.0**	**201.7**	**46.3**	**121.3**	**89**
**SD**	**11**	**1.3**	**1.7**		**108.4**	**22.3**	**16**	**16.9**	**1.8**
**Mean**†	**32.9**	**25.6**	**2.1**	**0**	**107.9**	**164.2**	**39.5**	**103.1**	**86.54.4**
**SD**	**9.6**	**2.9**	**1.8**		**60**	**16.6**	**7.4**	**18.1**	

BMI – body mass index, Kg/m^2^; AHI – apnea hypopnea index; %TO_2_<90% - Percent time asleep with arterial oxygen saturation below 90%; TG - Triglycerides; Cholest.- Cholesterol; * - current smoker; # - irregular low-dose acetyl salicylic acid usage (micropirin-75); Individual data for obstructive sleep apnea (OSA) patients and 3 control subjects (cont.) which underwent full-night polysomnography (Technion Sleep Medicine Center, Haifa). Blood samples were withdrawn under fasting after polysomnography. † Mean ± SD of control subjects in which disordered breathing during sleep was ruled out by a validated home monitored device.

**Figure 5:**
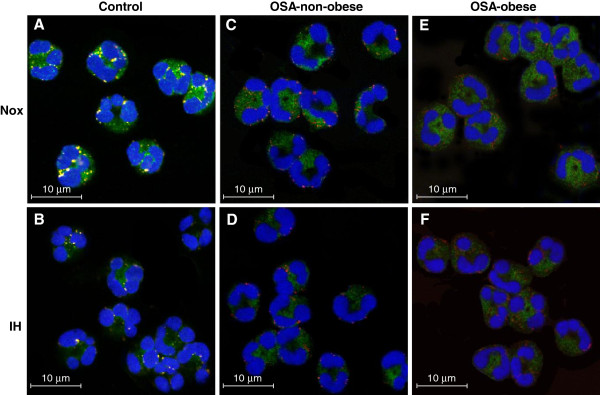
**Bax expression and co-localization with mitochondria in neutrophils of OSA patients and controls.** Bax expression and co-localization with mitochondria was analyzed by confocal laser scanning microscopy in neutrophils of OSA patients and controls in normoxia (Nox) and intermittent hypoxia (IH). The cytoplasmic distribution of mitochondria (red fluorescence) and Bax (green fluorescence) was determined by double immunofluorescence labeling as described in methods. (**A**, **B**) Representative images of a control subject (age=38 yr, BMI=25.3 Kg/m^2^, AHI=6.0 events/hrs, CRP=1.48mg/L, 19% apoptotic neutrophils were detected by Giemsa staining). (**A**) After 6 hrs of normoxia (Nox) Bax is translocated to and colocalized with mitochondria (yellow and orange). Manders Overlap Coefficient (MOC) >0.6 in neutrophils, (**B**) IH *in*-*vitro* inhibited Bax translocation to the mitochondria. (**C**) Representative images of a non-obese OSA patient (age=37 yr, BMI=24.7 Kg/m^2^, AHI=35.0 events/hrs, CRP=1.99 mg/L, 8% apoptotic neutrophils were detected by Giemsa staining) in normoxia and (**D**) in IH *in*-*vitro*. (**E**) Representative images of an obese OSA patient (age=48 yr, BMI=34.2 Kg/m^2^, AHI=33.0 events/hrs, CRP=8.09 mg/L, 9% apoptotic neutrophils were detected by Giemsa staining) in normoxia and in (**F**) IH *in-vitro*. In obese and non-obese OSA patients Bax was not translocated to the mitochondria; Bax (green) and mitochondria (red) were located separately in normoxia (**C**, **E**) and in IH (**D**, **F**).

The pre-apoptotic neutrophils of these control subjects expressed Bax translocation to the mitochondria under normoxia as described earlier for healthy controls (Figure 5A), and treatment with IH inhibited Bax/mitochondria co-localization (Figure 5B). In contrast, in patients with OSA there was little, if any, Bax translocation and co-localization to the mitochondria in normoxia (Figure 5C,E), as well as in IH (Figure 5D, F). These findings were noted in non-obese patients with low CRP levels (Figure 5C, D) as well as in obese patients with high CRP levels (Figure 5E,F).

As stated above, the fluorescence intensity of Bax and Mcl-1 expression was an individual trait. We therefore used Bax/Mcl-1 ratio for comparing the redistribution of pro-/anti-apoptotic proteins between OSA and healthy controls. The average Bax/Mcl-1 ratio in normoxia was 2-fold higher in healthy controls as compared to OSA patients and was significantly decreased by about 60% and 50% after treatment with IH and SH, respectively (Table 2). In OSA patients, the Bax/Mcl-1 ratio was already low at normoxia (1.0±0.5) and was further decreased after exposure to IH as depicted in Table 2. Similar values were obtained for Bax/Mcl-1 ratio in normoxia immediately after harvesting the cells (data not shown).

**Table 2:** Bax/Mcl-1 ratio in controls and OSA patients

**Treatment groups**	**Nox (6 hrs)**	**IH (6 cycles)**	**SH (6 hrs)**	**P1**	**P2**
Controls (n=5)	1.97 ± 0.7	0.8 ± 0.5	1.05± 0.6	0.002†	0.04
Controls* (n=3)	2.0 ± 0.4	1.14 ± 0.4	ND	0.09	
Controls pooled (n=8)	1.99 ± 0.3	0.97 ± 0.2	ND	0.001	
OSA (n=7)	1.0 ± 0.5‡	0.63 ± 0.3	ND	0.09	

Normoxia (Nox), intermittent hypoxia (IH), sustained hypoxia (SH).

* - Healthy controls were investigated in parallel with OSA patients in the Sleep Lab.

ND – not determined. Values represent means ± SD. P1 - statistical significance, IH vs. Normoxia (Nox); P2 - statistical significance, SH vs. Nox. †Once Bonferroni correction was added to compare the effects of IH and SH vs. Nox (for multiple comparisons, values of p<0.017 were considered significant), only the effects of IH remained significant.

‡−Differences between OSA patients and controls under normoxic conditions, p=0.015. A paired two-tailed *t test* was used for a single comparison.

## Discussion

Neutrophils survival was shown to increase in response to IH *in-vitro* as well as *in-vivo*[5,9], however, the underlying mechanisms are not entirely understood. In the present study we investigated the contribution of the mitochondrial stress-induced pathway in prolonging neutrophil survival under IH treatment *in-vitro* and in a human IH model *in-vivo*. In neutrophils treated by IH *in-vitro* the expression of the pro-apoptotic protein Bax was decreased, Bax translocation to the mitochondria was inhibited and the anti-apoptotic protein Mcl-1 was up-regulated via activation of ERK1/2 and p38MAPK dependent signaling pathways. In SH treated neutrophils, unlike in IH, Mcl-1 up-regulation was only dependent on p38MAPK but not on ERK1/2 activation. Moreover, using a quantitative confocal microscopy analysis we have shown that the hypoxia-induced changes in Bax/Mcl-1 expression and translocation were noted in neutrophils before the appearance of apoptotic morphology. Similarly to the *in-vitro* findings, in OSA patients undergoing nightly IH, Bax did not co-localize with the mitochondria and Bax/Mcl-1 ratio was significantly lower than in healthy controls.

The Bcl-2 family of proteins is one of the key regulators of cell death at the mitochondrial level [17,19,46-49], and Bax is the best known pro-apoptotic protein. In most cell types, the expression and activity of anti-apoptotic Bcl-2 members is higher than pro-apoptotic members. By contrast, mature neutrophils constitutively express pro-apoptotic proteins and the expression of the anti-apoptotic Bcl-2 members is very low [11,50]. Thus, the balance between pro- and anti-apoptotic members determines the fate of cells [51].

High Bax expression and its fusion with mitochondria were noted in apoptotic neutrophils by confocal microscopy analysis. Bax was also abundantly expressed, to a lower extent, in normoxic neutrophils of healthy subjects. However, its expression and translocation to the mitochondria were significantly lowered under IH as well as SH *invitro.* Although the critical molecules which inhibit Bax translocation to the mitochondria are as yet unknown, a possible candidate might be Mcl-1, which was up-regulated by nearly 2-fold under IH and SH. In freshly isolated neutrophils, Mcl-1 is heterodimerized in the cytoplasm with Bax. Diminishing Mcl-1 levels release Bax from the heterocomplex Bax:Mcl-1, and allow Bax to translocate to the mitochondria where it can exercise its pro-apoptotic function [13,26,46,52]. Its translocation to the mitochondria leads to the release of pro-apoptotic factors such as cytochrome *c*, which complexes with apoptotic protease-activating factor 1 (Apaf-1) and pro-caspase-9 to form a protein complex the ‘apoptosome’ which is involved in caspase-3 activation. The latter is responsible for the visible signs of apoptosis [16,19,48,51,52]. Accordingly, our findings demonstrate that changes in Bax/Mcl-1 expression and translocation to the mitochondria were noted before the appearance of apoptotic morphology, as expected, but also before caspase activation, as indicated by flow cytometry and confocal microscopy using FAN-FLICA Poly Caspase Kit (binds the activated caspases-1, -3, -4, -5, -6, -7, -8, -9) (Dyugovskaya L, Berger S, unpublished observation).

Mcl-1 has a short half-life (~3 hrs), and spontaneous apoptosis is accompanied by Mcl-1 degradation [31]. However, its expression may be increased depending on the stimuli exerted [15]. Mcl-1 expression was increased in IH and SH *in-vitro* treated neutrophils compared to normoxia. It is likely that both IH and SH may induce Mcl-1 stabilization by preventing its degradation, and/or possibly by up-regulating its production. Besides in IH and SH demonstrated in this study, increased Mcl-1 levels have been previously implicated in neutrophil survival induced by LPS, cytokines such as GM-CSF, IL-1, TNF-α, IL-15 [24-27,29], leukotriene B4 [28], Toll-like-receptor agonist [53] and SH for at least 8 hrs at less than 2% oxygen [31], as obtained in this study for 6 hrs of SH.

Mcl-1 expression in neutrophils is regulated by a diverse array of signal transduction pathways which depend on the stimuli exerted [25,44]. Here, we demonstrate for the first time that only under IH the up-regulation of Mcl-1 coincided with p-ERK1/2 activation, and by inhibiting ERK1/2, the expression of Mcl-1 was inhibited. In contrast, p38MAPK was up-regulated by both IH as well as by SH as previously shown [9], and its inhibition affected Mcl-1 expression under both hypoxic conditions. Also, like in our SH experimental conditions, similar findings were reported for neutrophils exposed to 12 hrs of SH. Inhibition of p38MAPK led to a significant decrease in Mcl-1 expression, whereas inhibiting ERK1/2 led only to a slight, but not significant decrease in Mcl-1 levels [30].

The selective ERK1/2 phosphorylation in human neutrophils by IH suggests that Mcl-1 activity might be regulated by different signal transduction pathways in various hypoxic conditions, such as in IH and SH demonstrated here. We should note however, that other pathways not investigated in this study, in addition to p38MAPK and ERK1/2 could be involved in the up-regulation of Mcl-1 under IH. For instance, the NF-κB-dependent up-regulation of IL-8 levels described earlier for IH [9] may control the expression of survival genes of Bcl-2 family members [54] by increasing anti-apoptotic and decreasing pro-apoptotic proteins levels in neutrophils [55].

Finally we showed for the first time that in OSA patients Bax translocation to the mitochondria was minimal in neutrophils maintained at normoxic conditions, and it was further reduced in response to IH *in-vitro* in all patients investigated regardless of weight differences. Moreover, the normoxic values obtained for OSA were similar to those of control neutrophils exposed to IH *in-vitro*, illustrating the similarities between *in-vitro* and *in-vivo* IH*.* Additionally, the ratio Bax/Mcl-1 was significantly lower in OSA patients at normoxia as compared to control subjects clearly demonstrating that pro-apoptotic Bax was low whereas the anti-apoptotic Mcl-1 protein was high. Collectively, these finding suggest that the IH-dependent prolonged neutrophil survival in OSA is largely affected by the mitochondrial stress-induced pathway.

Elucidating potential mechanisms which might suppress neutrophil apoptosis by IH *in-vivo*, is of a great importance to OSA and sleep disordered breathing (SDB). OSA is a prevalent syndrome associated with cardiovascular morbidity and mortality [56]. It affects at least 4% and 2% of men and women in the adult population. However, the prevalence of SDB (IH without the characteristic excessive daytime sleepiness) is estimated to be as high as 24% and 9% in men and women. This value may rise to 60-90% in obese individuals [57]. Moreover, SDB is also highly prevalent in more than 60% of patients with acute myocardial infarction and in more than 50% (44-93%) of patients with stroke [56]. Furthermore, OSA and SDB are also associated with increased vascular inflammation, endothelial dysfunction and atherosclerosis [56,58].

### Study limitations

The limitations of our study regarding the patients with OSA should be acknowledged. First, the IH experienced by OSA patients was not mimicked in our IH *in-vitro* model due to technical constrains. The short intervals as experienced by patients with OSA are rather difficult to reproduce with neutrophils in culture conditions because they are non-adherent cells. Therefore, the medium cannot be replaced at short intervals with alternating preconditioned hypoxic or normoxic medium. Despite that, the findings in OSA patients were similar to those obtained by IH *in-vitro.* Yet, it would be interesting to repeat this type of a study in an animal model of IH, that allows mimicking the time patterns of IH more closely to OSA patients and in a dose dependent manner, and to avoid confounding factors and comorbidities [59].

Second, the number of OSA patients investigated in the current study is relatively small and there are significant differences in age and BMI between patients and controls. However, in a previous study [5] meticulously and rigorously investigating more than 100 patients we have shown by linear regression analysis that all apnea measures (AHI, oxygen desaturation index of 3%, minimal oxygen desaturation, and % time spent below 90% saturation) were significantly correlated with decreased neutrophil apoptosis, clearly attesting to the importance of OSA severity. Moreover, using multivariate analysis we could verify that this relationship was independent of BMI, age, CRP, triglycerides, and adiponectin. Thus, although some of the patients investigated in the present study were obese and had higher CRP levels than controls, their results with respect to Bax/Mcl-1 ratio and the lack of Bax translocation to the mitochondria were identical to the results obtained in non-obese OSA patients with low CRP levels and like in the IH *in-vitro,* and unlike the normoxic controls’ findings. It is noteworthy that in the previous study [5] we also demonstrated the contribution of the IH and the AHI severity measures, regardless of confounding factors, in attenuating neutrophil apoptosis by investigating OSA patients with and without treatment with nasal continues positive airway pressure (nCPAP), which ameliorates the apneas. Patients on nCPAP treatment were enrolled and were investigated on two consecutive nights, one with and one without nCPAP. Patients demographics, BMI, lipid profile or CRP were unaffected by omitting the treatment, yet all apnea severity measures were increased and neutrophil apoptosis was decreased as compared to the treatment night. These earlier data clearly emphasize the role played by the IH in increasing neutrophils lifespan, therefore allowing to investigate the mechanisms of neutrophil apoptosis under conditions of IH *in-vivo* in patients as well as *in-vitro* in healthy controls.

## Conclusions

This study demonstrates that Bax/Mcl-1 ratio was significantly lowered in neutrophils treated by IH *in-vitro* and in patients with OSA by up-regulating the anti-apoptotic Mcl-1 and down-regulating the pro-apoptotic Bax. As a result of the IH, Bax translocation to the mitochondria was prevented. Thus, IH converts the pro-apoptotic phenotype into an anti-apoptotic one by modulating the Bcl-2 family members Bax and Mcl-1. This effect of IH is specifically mediated through ERK1/2 and p38MAPK signaling pathways whereas in SH it is mediated only through p38MAPK. Hence, identifying neutrophil survival pathways affected by IH may lead to new approaches in treating some sleep apnea complications associated with endothelial dysfunction and atherosclerosis. Moreover, these findings may bear relevance to other conditions and co-morbidities associated with components of IH such as physical activity, brief ascents to altitude, myocardial infarction and cancer.

## Abbreviations

(OSA): Obstructive sleep apnea; (SDB): Sleep disordered breathing; (IH): Intermittent hypoxia; (SH): Sustained hypoxia; (GM-CSF): Granulocyte-macrophage colony-stimulating factor; (AHI): Apnea-hypopnea index; (PMNs): Polymononuclear cells; (Bcl): B-cell lymphocytic-leukaemia proto-oncogene; (Bax): Bcl-2 associated X protein; (Mcl-1): Myeloid cell leukemia 1, also termed myeloid cell factor-1; (MOC): Manders overlap coefficient.

## Competing interests

The authors declare that they have no competing interests.

## Authors’ contributions

All authors have read and approved the final manuscript. The special contributions of each author are: Conception and design: LD, LL; Acquisition of data LD, LL, PL, AP, VC; Analysis and interpretation: LD, AP, PL, LL; Drafting the manuscript for important intellectual content: LL, LD AP, LP, VC; Approval of the manuscript LL, LD AP, LP, VC.
